# Quantitative evaluation of vertical control in orthodontic camouflage treatment for skeletal class II with hyperdivergent facial type

**DOI:** 10.1186/s13005-024-00432-2

**Published:** 2024-05-14

**Authors:** Yan-Ning Guo, Sheng-Jie Cui, Ye Liu, Yu Fu, Jie-Ni Zhang, Yan-Heng Zhou, Xue-Dong Wang

**Affiliations:** 1 Department of Orthodontics, National Center of Stomatology & National Clinical Research Center for Oral Diseases & National Engineering Laboratory for Digital and Material Technology of Stomatology & Beijing Key Laboratory of Digital Stomatology & Research Center of Engineering and Technology for Computerized Dentistry Ministry of Health & NMPA Key Laboratory for Dental Materials, Peking University School and Hospital of Stomatology, No.22, Zhongguancun South Avenue, Haidian District, Beijing, 100081 China; 2 Dental Medical Center, China-Japan Friendship Hospital, Beijing, 100029 China; 3 Department of Orthodontics, the School of Stomatology, The Key Laboratory of Stomatology, Hebei Medical University, Shijiazhuang, 050017 China; 4 Fourth Division Department, National Center of Stomatology & National Clinical Research Center for Oral Diseases & National Engineering Laboratory for Digital and Material Technology of Stomatology & Beijing Key Laboratory of Digital Stomatology & Research Center of Engineering and Technology for Computerized Dentistry Ministry of Health & NMPA Key Laboratory for Dental Materials, Peking University School and Hospital of Stomatology, Beijing, 100081 China

**Keywords:** Skeletal class II, Vertical control, Profile improvement

## Abstract

**Background:**

In this study, we sought to quantify the influence of vertical control assisted by a temporary anchorage device (TAD) on orthodontic treatment efficacy for skeletal class II patients with a hyperdivergent facial type and probe into the critical factors of profile improvement.

**Methods:**

A total of 36 adult patients with skeletal class II and a hyperdivergent facial type were included in this retrospective case–control study. To exclude the effect of sagittal anchorage reinforcement, the patients were divided into two groups: a maxillary maximum anchorage (MMA) group (*N* = 17), in which TADs were only used to help with anterior tooth retraction, and the MMA with vertical control (MMA + VC) group (*N* = 19), for which TADs were also used to intrude the maxillary molars and incisors. The treatment outcome was evaluated using dental, skeletal, and soft-tissue-related parameters via a cephalometric analysis and cast superimposition.

**Results:**

A significant decrease in ANB (*P* < 0.05 for both groups), the retraction and uprighting of the maxillary and mandibular incisors, and the retraction of protruded upper and lower lips were observed in both groups. Moreover, a significant intrusion of the maxillary molars was observed via the cephalometric analysis (− 1.56 ± 1.52 mm, *P* < 0.05) and cast superimposition (− 2.25 ± 1.03 mm, *P* < 0.05) of the MMA + VC group but not the MMA group, which resulted in a remarkable decrease in the mandibular plane angle (− 1.82 ± 1.38°, *P* < 0.05). The Z angle (15.25 ± 5.30°, *P* < 0.05) and Chin thickness (− 0.97 ± 0.45°, *P* < 0.05) also improved dramatically in the MMA + VC group, indicating a better profile and a relaxed mentalis. Multivariate regression showed that the improvement in the soft tissue was closely related to the counterclockwise rotation of the mandible plane (*P* < 0.05).

**Conclusions:**

TAD-assisted vertical control can achieve intrusion of approximately 2 mm for the upper first molars and induce mandibular counterclockwise rotation of approximately 1.8°. Moreover, it is especially important for patients without sufficient retraction of the upper incisors or a satisfactory chin shape.

**Supplementary Information:**

The online version contains supplementary material available at 10.1186/s13005-024-00432-2.

## Background

For adult patients with severe class II malocclusion accompanied by a hyperdivergent growth pattern, orthognathic surgery is usually the optimal therapy to improve facial aesthetics and masticatory function [1, 2]. Nevertheless, some patients refuse surgery due to its possible risks and high cost. Orthodontic camouflage treatment provides an alternative for such patients [3, 4].

To improve the profile of this kind of patient, both sagittal retraction and vertical control are important. Several studies have found and confirmed the importance of vertical control in orthodontic treatment for skeletal class II malocclusion [5–7]. However, varying treatment methods are used. For adolescent patients, the most effective approach is often to utilize their vertical growth potential to guide their facial development in the desired direction. Jamilian et al. applied a modified functional orthodontic appliance to induce sagittal and vertical changes in the mandible, achieving significant facial improvement for a patient with severe skeletal class II [8].

On the other hand, for adult patients lacking growth potential, active intrusion of posterior teeth is required to intervene vertically. Early on, high-pull headgear was the most common vertical control method, but this approach relied heavily on patient compliance, and it involved the application of intermittent force, making it relatively unreliable [9–11].

TADs’ emergence has greatly improved the convenience and efficiency of treatment [12, 13]. Compared to headgear, TAD-assisted vertical control can provide more dental intrusion and counterclockwise rotation of the mandibular plane, which contributes to further improvement of profile [14, 15]. Additionally, when active intrusion was applied, we typically utilize a sustained light force (approximately 50 g), which is more favorable for the remodeling of periodontal tissues compared to the intermittent heavy force exerted by headgear.

However, the mini-implants placed in the maxilla’s posterior region can also provide strong sagittal anchorage. Several studies have shown that maximum anchorage itself can achieve a better treatment outcome and improve the profile [16–18]. These findings have prompted the following questions: If sagittal retraction can already lead to sufficient facial aesthetics, is vertical tooth movement still necessary? To what extent can vertical movements benefit the facial profile?

Our research group has paid close attention to the efficacy of TAD-assisted vertical control in orthodontic camouflage treatments for patients with skeletal class II malocclusion. We have published several case reports and long-term follow-up studies showing that vertical control significantly improved the profiles of patients with skeletal class II malocclusion and a hyperdivergent facial type, achieving good long-term stability [19–23]. We believe that specifying how the active intrusion of upper dentition contributes to these craniofacial improvements will provide more information about the ability and limits of TAD-assisted vertical control and broaden the understanding of orthodontic camouflage treatment. Therefore, we included a control group whose TADs were used only to reinforce maxillary sagittal anchorage in order to exclude the influence of sagittal retraction.

With this retrospective case–control study, we aimed to quantify the effectiveness of TAD-assisted vertical control in the improvement of dentoalveolar malformation and soft tissue profiles in adult patients with a severe skeletal class II hyperdivergent pattern, and justified the necessity of active intrusion. We believe that this article provides specific references for orthodontists and general dentists concerning the camouflage treatment of patients with skeletal class II malocclusion.

## Methods

This study was based on retrospective data obtained from orthodontic records at the Peking University School and Hospital of Stomatology, and it was approved by the institution’s biomedical ethics committee (approval number: PKUSSIRB-201630096, retrospectively registered). The patients included in this study accepted orthodontic treatment between 2006 and 2018.

The study’s sample selection was based on the following inclusion criteria: good-quality orthodontic records, the presence of permanent dentition, age > 18 years, a convex profile, skeletal class II (ANB > 5°), and a hyperdivergent skeletal pattern (FMA > 28°) [24]. The exclusion criteria included the following: dental anomalies in size, number, shape, or structure; permanent tooth loss; orthodontic–orthognathic combined surgery treatment; and Botox injection or prosthesis implantation before or during orthodontic treatment.

### Treatment protocols

All the participants underwent systematic periodontal and endodontic assessments and therapies before orthodontic interventions. A straight-wire MBT technique was utilized after the extraction of four premolars from all patients. Braces and archwires were obtained from TP Orthodontics (La Porte, IN, USA). The alignment and leveling phases involved initial bracket-bonding followed by a certain procedure utilizing 0.014 in. NiTi, 0.016 in. NiTi, 0.016 in. × 0.022 in. NiTi, and 0.019 in. × 0.025 in. NiTi archwires sequentially. During the space-closing phase, a 0.019 × 0.025 in. stainless steel archwire was applied using a conventional sliding mechanism. This phase was terminated upon the complete closure of the premolar spaces. The patients’ dentition was finely adjusted before debonding. Miniscrews (diameter: 1.5 mm; length: 7 mm; Zhongbang Medical Treatment Appliance, Xi’an, China) were surgically inserted into the alveolar ridge.

**Fig. 1:**
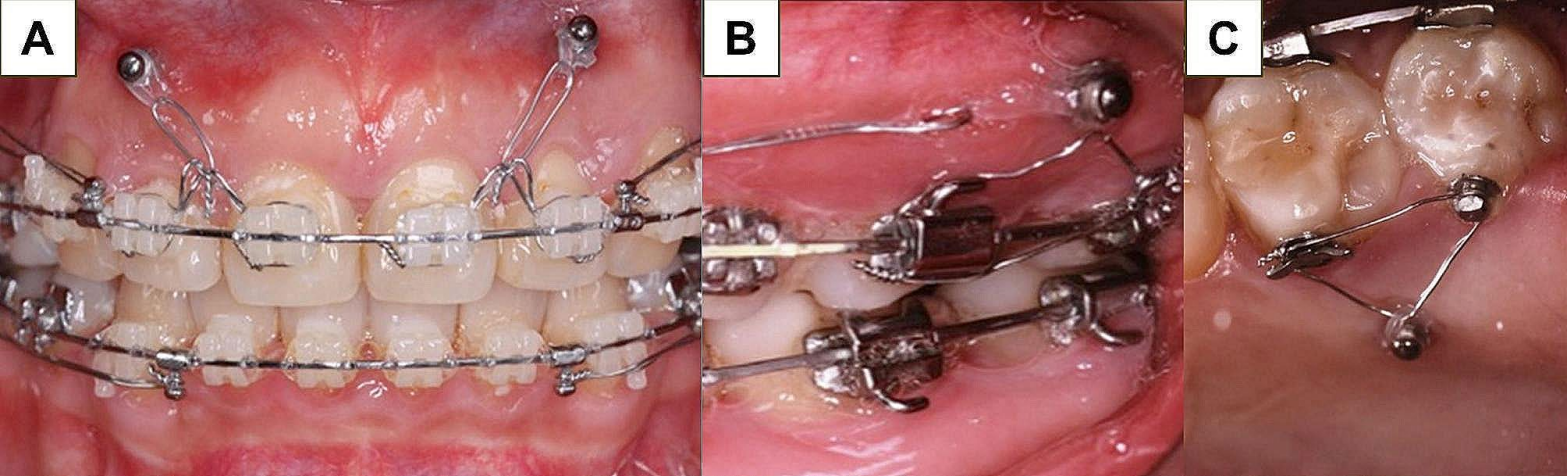
Representative image of intraoral devices. **A**. TAD-assisted intrusion of the upper anterior teeth. **B**. Buccal view of the posterior intrusion devices. **C**. Palatal view of the posterior intrusion devices

The patients were divided into two groups: (1) the maxillary maximum anchorage (MMA) group, in which TADs were implanted only at the bilateral buccal side of the alveolar bone, between the roots of the upper premolar and the upper first molar or between the upper first molar and the upper second molar; and (2) the maxillary maximum anchorage with vertical control (MMA + VC) group, in which TADs were implanted into the bilateral buccal and lingual sides of the alveolar bone, between the roots of the upper first molar and the upper second molar, to intrude the upper molars with or without the TADs implanted in the anterior segment for incisor intrusion (Fig. 1).

### Sample size calculation

In this study, the effect size of the primary outcome was expected to be 2.32. This number was the difference in mandibular counterclockwise rotation (the decrease in the FMA value) between the two groups calculated in our preliminary study. The sample size was calculated using online software (http://hedwig.mgh.harvard.edu/sample_size/) by assuming 5% type I errors and 20% type II errors. The sample calculation indicated that at least 10 patients were needed in each group.

In total, 36 patients were selected for the current study. The MMA group comprised 17 patients (14 females, 3 males) with a mean age of 24.18 ± 3.83 years and a mean treatment duration of 34.4 ± 12.8 months. The MMA + VC group consisted of 19 patients (16 females, 3 males) aged 25.00 ± 4.99 years, whose mean treatment duration was 34.7 ± 6.8 months. No significant difference in the patients’ gender, age, or treatment duration was observed between the groups (Additional Table 1).

### Cephalometric analysis

**Fig. 2:**
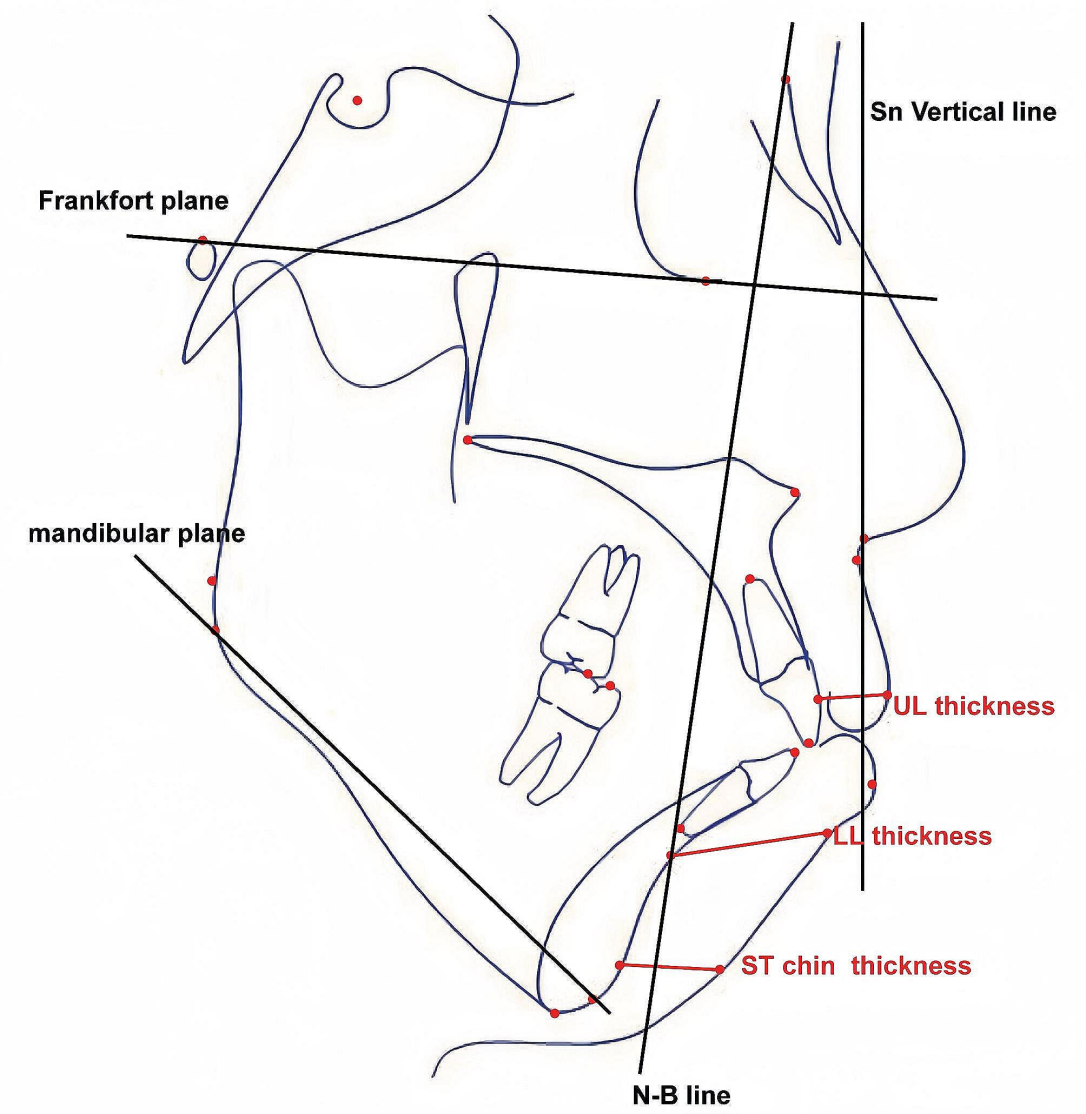
Tracing of a pretreatment cephalometric radiograph

Pre-treatment and post-treatment lateral cephalograms were collected, digitized, and superimposed using the Dolphin 11.0 software (Dolphin Imaging, Chatsworth, CA). An investigator who was not informed about the study’s groups obtained the measurements, which a second blinded investigator checked for accuracy. Any disagreements between these investigators were resolved through a weighted reevaluation until they were both satisfied. The variables used in the cephalometric analysis included skeletal, dental, and soft-tissue-related measurements. In total, 29 such variables were used (8 skeletal, 12 dental, and 9 soft-tissue-related). Figure 2 depicts the landmarks and important variables used in this study, while Additional Table 2 provides definitions.

### Dental cast analysis

**Fig. 3:**
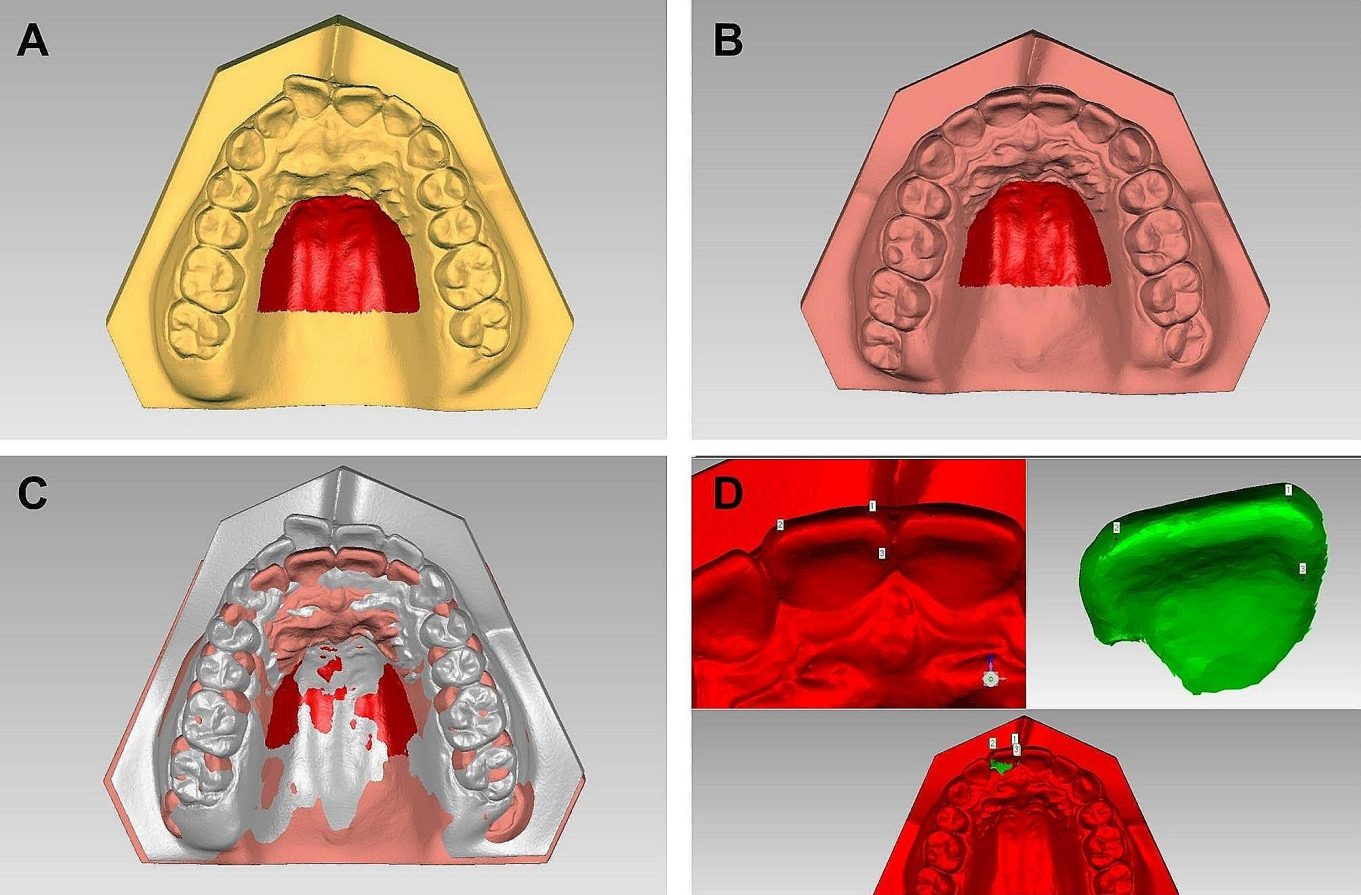
Superimposition of the dental casts. **A**. The pre-treatment maxillary model. **B**. The post-treatment maxillary model. **C**. Superimposition based on the stable structure of the palate. **D**. Transfer of corresponding landmarks

Pre-treatment and post-treatment dental casts were scanned using a 3Shape scanner (3Shape D, Kopenhagen, Dänemark) and measured in a double-blinded manner by a trained orthodontist using the Geomagic 13.0 software (Geomagic Qualify, Durham, NC, US). As Fig. 3 shows, the superimposition of the dental casts was based on the palate’s stable structure. A coordinate system was built, based on the definition of the anatomical occlusal plane and the midline of the palate. The tooth movements were analyzed in two dimensions, anterior or posterior (X) and intrusion or extrusion (Z). Additionally, posterior and extrusive movement was defined as positive.

To evaluate the method’s error, 10 post-treatment lateral cephalograms and digital casts were randomly selected and remeasured by the same examiners two weeks after the first measurement was obtained. The intraclass correlation coefficient (ICC) was used to assess intra-examiner reliability and the reproducibility of all linear and angular measurements.

### Statistical analysis

The intraclass correlation efficient (ICC) was evaluated using a two-way random model. Descriptive statistics for the dental casts and radiographic measurements were calculated for both the first and second measurements. Comparisons were performed and correlations were identified using Student’s *t* test in accordance with the results of Shapiro–Wilk normality tests. The pre-treatment skeletal, dental, and soft-tissue-related variables were compared between the groups using independent-sample *t* tests. The same variables were also compared from pre-treatment to post-treatment using paired *t* tests. The differences in treatment changes (concerning both the lateral cephalograms and the dental casts) between the MMA and MMA + VC groups were evaluated using independent-sample *t* tests. Multiple linear regression analysis was used to test the correlation between the independent variables of craniofacial structures and the dependent variable, the Z angle. Both groups’ differences in treatment changes were normalized to the mean variance. Then, a backward method was used to screen the independent variables. The entry probability of *F* was 0.05, and the removal criterion was 0.1. The statistical tests were performed with SPSS 18.0 software (IBM Corp., Armonk, NY). The results were considered statistically significant at *P* < 0.05.

## Results

The groups were similar in age at the beginning of the orthodontic treatment (Additional Table 1). ICC was calculated with good reproducibility of the measurements (0.810–0.997), as Additional Table 3 shows.

The two groups showed similar mandibular retrognathia and hyperdivergent skeletal patterns. However, differences were observed in several variables, such as the Z angle, ANB, and L1-NB (mm). These differences indicated that the patients in the MMA + VC group had a more convex profile and more severe malocclusion (Table 1).

**Table 1:** Pre-treatment severity of skeletal mandibular retrognathia and hyperdivergency of the MMA and MMA + VC groups

Variables	MMA (*N* = 17)		MMA + VC (*N* = 19)		*P*
	Mean	SD	Mean	SD	
Skeletal					
SNA (°)	82.82	3.04	84.06	2.92	0.219
SNB (°)	76.06	2.91	75.91	3.34	0.884
ANB (°)	6.75	0.79	8.22	1.43	0.001*
MP-SN (°)	42.27	3.90	44.47	5.38	0.174
FMA (°)	33.27	3.38	35.86	5.75	0.114
PFH/AFH (%)	44.88	3.78	43.84	3.03	0.367
Pog-NB (mm)	-0.30	1.43	-1.06	1.49	0.128
ANS-Me (mm)	65.16	4.65	64.07	4.38	0.476
Dental					
U1 - NA (°)	20.51	7.07	20.84	7.62	0.896
U1 - SN (°)	103.26	7.32	104.89	7.83	0.525
L1 - NB (°)	34.78	5.29	37.32	5.01	0.149
IMPA (°)	96.59	4.96	96.94	5.10	0.837
Inter incisor angle (U1-L1) (°)	117.99	9.30	113.71	8.92	0.167
U1-NA (mm)	4.48	2.57	4.58	2.21	0.893
L1-NB (mm)	9.61	1.93	10.93	1.55	0.028*
U1-PP (mm)	30.70	2.80	30.25	2.56	0.620
U6-PP (mm)	20.21	2.48	19.98	2.59	0.791
L1-MP (mm)	42.96	3.25	43.48	2.34	0.582
L6-MP (mm)	32.65	3.10	31.88	2.66	0.429
Occlusal Plane(°)	12.85	3.64	12.18	4.56	0.631
Soft-tissue-related					
UL Angle-SnV (°)	12.11	5.33	16.69	6.23	0.024*
Z Angle (°)	58.89	4.70	49.39	6.94	< 0.001*
UL thickness (mm)	11.64	1.63	11.51	2.50	0.856
LL Thickness (mm)	14.36	2.87	14.86	2.14	0.556
Chin Thickness (mm)	12.36	2.66	13.71	2.48	0.126
ULA-Sn Vertical (mm)	3.58	1.41	4.72	1.66	0.034*
LLA-Sn Vertical (mm)	-0.09	3.43	0.66	2.21	0.438
UL Length (mm)	22.41	2.49	22.68	2.07	0.722
LL Length (mm)	44.62	3.28	42.92	4.02	0.173

### TAD-assisted vertical control better improved patients’ profiles

TADs’ efficacy in improving therapeutic outcomes is certain. However, whether and to what extent TAD-assisted vertical control can help patients with skeletal class II achieve better results from camouflage orthodontic treatments compared to the simple reinforcement of the maxillary anchorage is unclear.

**Fig. 4:**
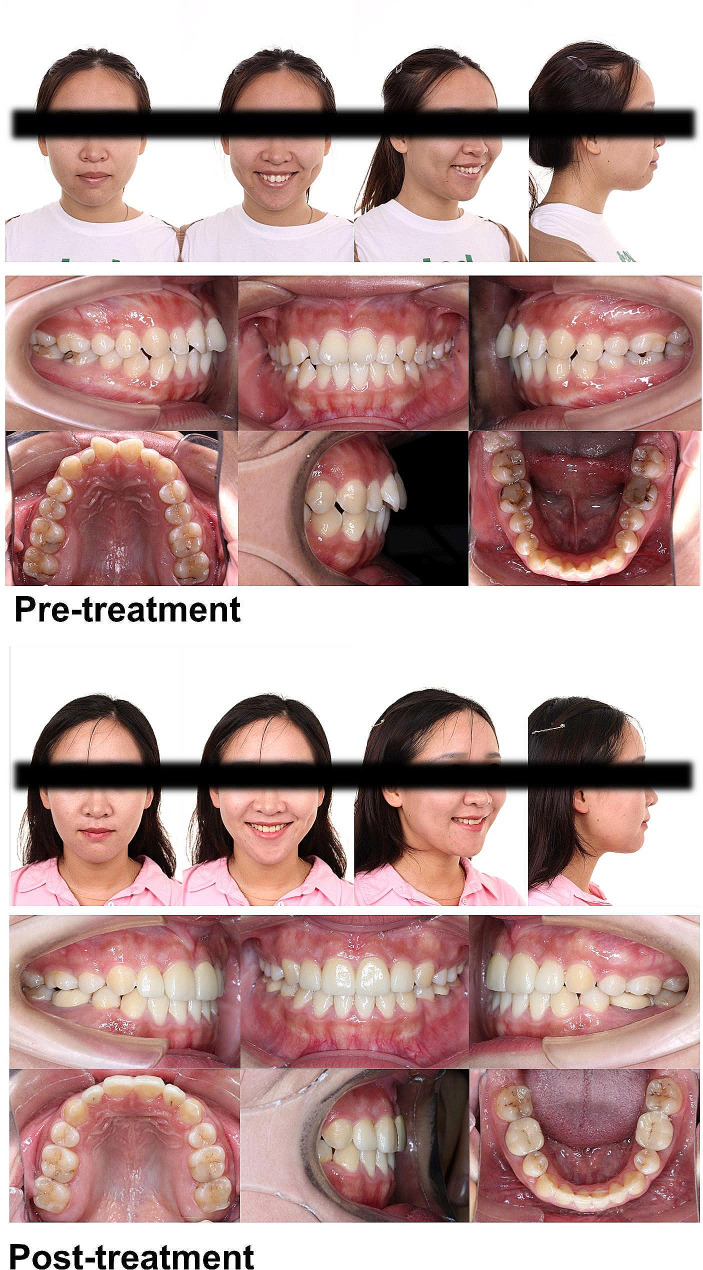
A representative case from the MMA group. The upper anterior incisors were restored using a ceramic veneer

**Fig. 5:**
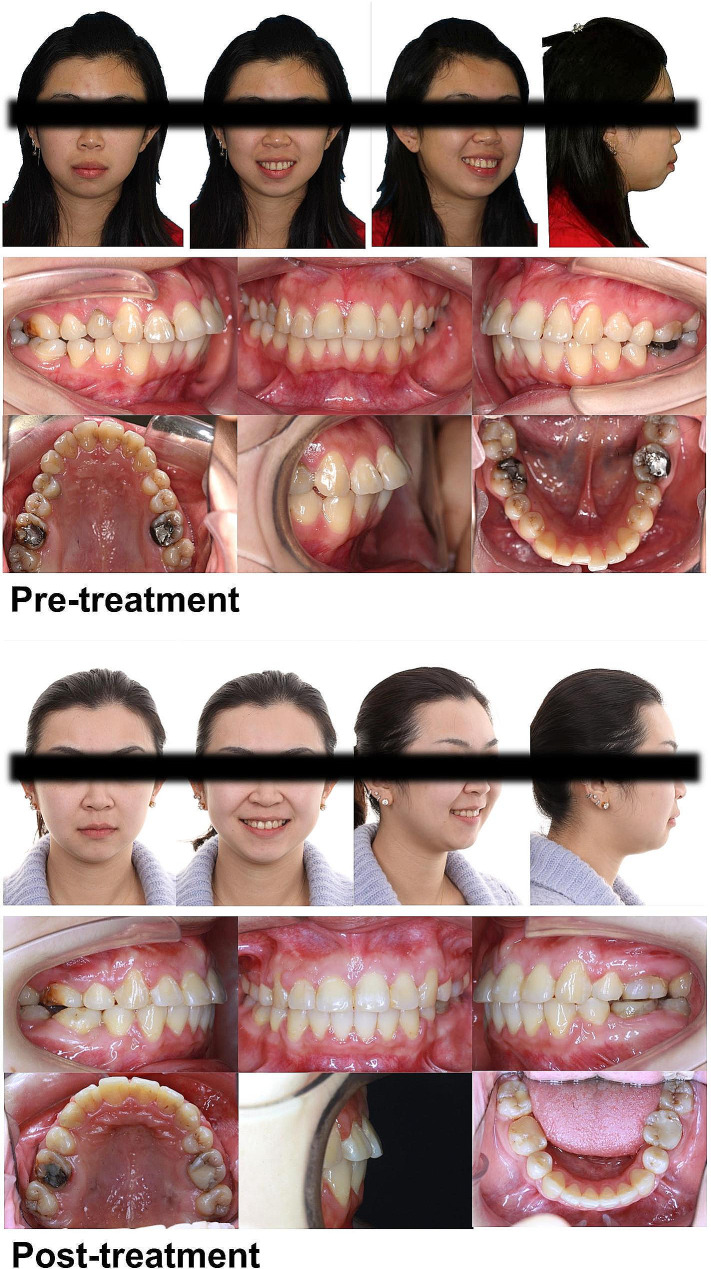
A representative case from the MMA + VC group

For most of the patients whose results we recorded, a convex profile was the main complaint. Therefore, we first analyzed the improvements in soft-tissue-related variables for both groups (Tables 2 and 3). We discovered a similar trend of lip retraction (the UL-SnV angle and distance and the LL-SnV distance) and soft tissue relaxation (UL thickness and LL thickness). However, the change in the Z angle and Chin thickness showed that patients in the MMA + VC group experienced more improvement in their profiles and mentalis relaxation. (Figures 4 and 5 show the representative cases of the two groups, respectively.) Through these results, we have shown that TAD-assisted vertical control further improved the patients’ profiles, but how this advantage was achieved remained unclear.

**Table 2:** Pre- and post-treatment comparison of soft-tissue-related variables through cephalometric analysis

Variables	MMA (*N* = 17)					MMA + VC (*N* = 19)				
	pre		post		*P*	pre		post		*P*
	Mean	SD	Mean	SD		Mean	SD	Mean	SD	
UL Angle-SnV (°)	12.11	5.33	6.82	5.34	0.009*	16.69	6.23	8.37	5.88	< 0.001*
Z Angle (°)	58.89	4.70	69.43	6.24	< 0.001*	49.39	6.94	64.64	8.79	< 0.001*
UL thickness (mm)	11.64	1.63	12.67	1.89	0.032*	11.51	2.50	13.06	2.32	< 0.001*
LL Thickness (mm)	14.36	2.87	12.30	2.12	0.004*	14.86	2.14	12.77	2.25	0.006*
Chin Thickness (mm)	12.36	2.66	12.64	2.12	0.597	13.71	2.48	12.73	2.17	0.009*
UL-Sn Vertical (mm)	3.58	1.41	1.86	1.34	0.001*	4.72	1.66	2.59	1.71	< 0.001*
LL-Sn Vertical (mm)	-0.09	3.43	-2.19	2.20	0.016*	0.66	2.21	-2.46	2.28	< 0.001*
UL Length (mm)	22.41	2.49	22.52	1.85	0.797	22.68	2.07	22.68	2.39	1.000
LL Length (mm)	44.62	3.28	45.29	3.61	0.316	42.92	4.02	44.14	3.96	0.021*

**Table 3:** Comparison of changes in soft-tissue-related variables through cephalometric analysis between the MMA and MMA + VC groups

Variables	MMA (*N* = 17)		MMA + VC (*N* = 19)		*P*
	Mean	SD	Mean	SD	
UL Angle-SnV (°)	-5.29	7.27	-8.32	6.54	0.196
Z Angle (°)	10.54	5.11	15.25	5.30	0.011*
UL thickness (mm)	1.03	1.81	1.55	1.58	0.366
LL Thickness (mm)	-2.06	2.56	-2.09	2.91	0.979
Chin Thickness (mm)	0.28	2.16	-0.97	1.45	0.046*
ULA-Sn Vertical (mm)	-1.72	1.82	-2.13	1.70	0.487
LLA-Sn Vertical (mm)	-2.10	3.23	-3.12	2.46	0.290
UL Length (mm)	0.11	1.67	0.00	1.94	0.861
LL Length (mm)	0.67	2.67	1.22	2.11	0.495

### TAD-assisted vertical control contributed to maxillary retraction and mandibular counterclockwise rotation

Remarkable decreases in SNA and ANB were discovered in both groups. Furthermore, the decrease in ANB in the MMA + VC group was significantly greater compared to MMA group, showing effective maxillary retraction, which partly explained the dramatic change in the soft tissue (Tables 4 and 5).

**Table 4:** Pre- and post-treatment comparison of skeletal variables through cephalometric analysis

Variables	MMA (*N* = 17)						MMA + VC (*N* = 19)				
	pre		post		*P*		pre		post		*P*
	Mean	SD	Mean	SD			Mean	SD	Mean	SD	
SNA (°)	82.82	3.04	81.18	2.77		< 0.001*	84.06	2.92	81.64	2.58	< 0.001*
SNB (°)	76.06	2.91	76.09	3.02		0.921	75.91	3.34	75.88	3.04	0.911
ANB (°)	6.75	0.79	5.10	1.02		< 0.001*	8.22	1.43	5.74	1.65	< 0.001*
MP-SN (°)	42.27	3.90	42.11	4.15		0.528	44.47	5.38	42.65	4.96	< 0.001*
FMA (°)	33.27	3.38	33.01	3.22		0.476	35.86	5.75	33.83	5.39	< 0.001*
PFH/AFH (%)	44.88	3.78	45.15	3.38		0.568	43.84	3.03	45.47	3.30	< 0.001*
Pog-NB (mm)	-0.30	1.43	0.06	1.48		0.031*	-1.06	1.49	-0.56	1.28	0.008*
ANS-Me (mm)	65.16	4.65	65.42	4.35		0.526	64.07	4.38	63.89	5.72	0.798

**Table 5:** Comparison of the changes in skeletal variables through cephalometric analysis between the MMA and MMA + VC groups

Variables	MMA (*N* = 17)		MMA + VC (*N* = 19)		*P*
	Mean	SD	Mean	SD	
SNA (°)	-1.64	1.27	-2.43	1.18	0.061
SNB (°)	0.02	0.62	-0.03	1.01	0.881
ANB (°)	-1.65	1.30	-2.48	0.84	0.020*
MP-SN (°)	-0.16	1.05	-1.82	1.38	< 0.001*
FMA (°)	-0.26	1.49	-2.03	2.05	0.006*
PFH/AFH (%)	0.28	1.96	1.64	1.45	0.023*
Pog-NB (mm)	0.36	0.63	0.51	0.74	0.530
ANS-Me (mm)	0.26	1.68	-0.18	3.09	0.598

**Fig. 6:**
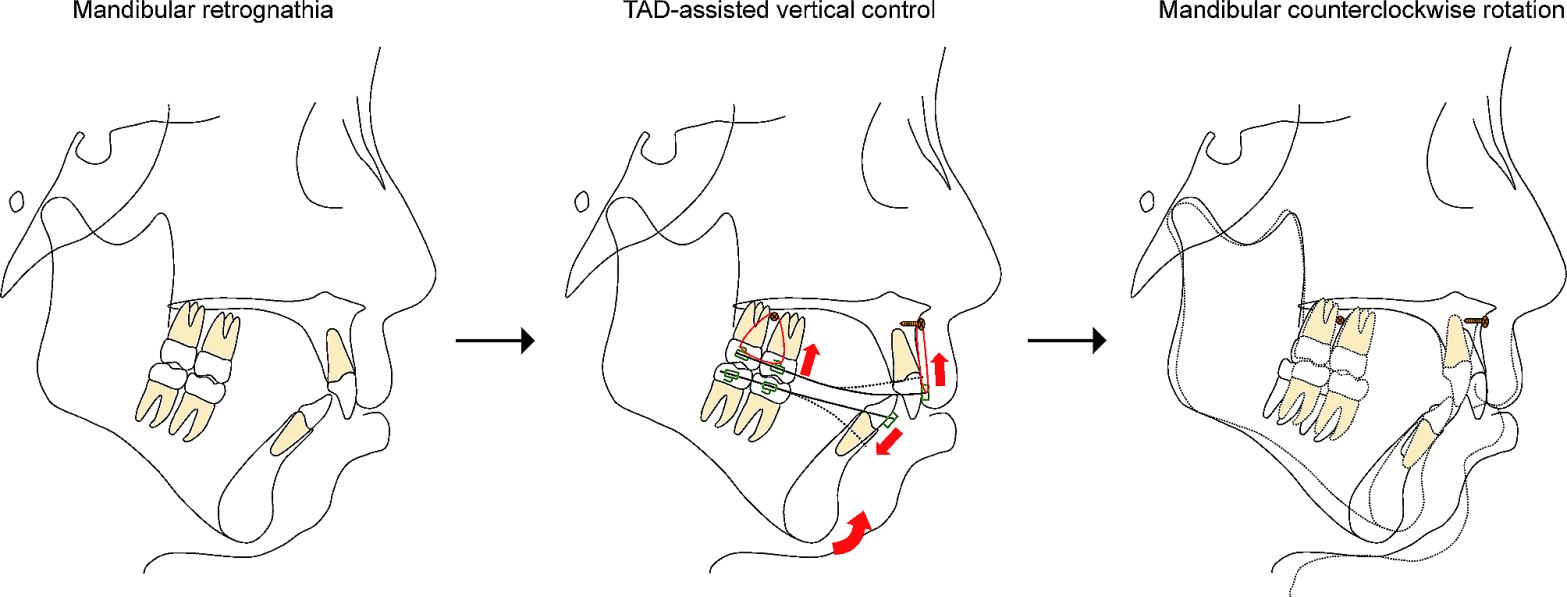
Schematic graph of TAD-assisted vertical control during orthodontic camouflage treatment for patients with skeletal class II and a hyperdivergent facial type

Additionally, no significant differences in the mandibular plane angle in the MMA group pre- and post-treatment were observed. Indeed, the lower facial height (ANS-Me) even increased slightly. Meanwhile, the MP-SN and FMA values significantly decreased in the MMA + VC group, suggesting that TAD-assisted vertical control effectively achieved mandibular counterclockwise rotation. The decrease in the mandibular plane angle showed a significant difference in the MP-SN and FMA values between the MMA and MMA + VC groups (Table 5). An emphatic change was also observed in the improvement of PFH/AFH, indicating an improvement in the hyperdivergent facial type. Thus, the application of TAD-assisted vertical control achieved a certain extent of mandibular counterclockwise rotation, which also helped improve patients’ profiles (Fig. 6).

### TADs achieved substantial vertical control via the intrusion of maxillary dentition

Despite the gratifying sagittal retraction of the incisors in both groups (Table 6), the study’s cephalometric analysis showed significant intrusion of the upper molar on the P-P plane (U6-PP) in the MMA + VC group but not in the MMA group. Similarly, the upper incisor showed more intrusion (U1-PP) in the MMA + VC group, though no significance was observed (Table 7). These results were confirmed via dental cast superimposition (Table 8). Compared to the MMA group, the MMA + VC group experienced significant intrusion of the upper dentition. However, our cephalometric analysis also revealed a significantly lower molar extrusion (L6-MP) on the mandibular plane in both groups during orthodontic treatment. Thus, the tooth movement in the vertical dimension manifested the intrusion of the upper dentition for the MMA + VC group and the extrusion of the lower molars for both groups.

**Table 6:** Pre- and post-treatment comparison of dental variables through cephalometric analysis

Variables	MMA (*N* = 17)					MMA + VC (*N* = 19)				
	pre		post		*P*	pre		post		*P*
	Mean	SD	Mean	SD		Mean	SD	Mean	SD	
U1 - NA (°)	20.51	7.07	14.92	4.59	0.017*	20.84	7.62	15.30	5.71	0.001*
U1 - SN (°)	104.22	7.81	96.98	6.60	0.003*	104.89	7.83	96.93	6.11	< 0.001*
L1 - NB (°)	34.78	5.29	26.06	5.62	< 0.001*	37.32	5.01	29.69	6.74	< 0.001*
IMPA (°)	96.59	4.96	87.86	6.39	< 0.001*	96.94	5.10	90.94	7.48	0.004*
Inter incisor angle (°)	117.99	9.30	133.91	7.66	< 0.001*	113.71	8.92	129.26	8.55	< 0.001*
U1-NA (mm)	4.48	2.57	1.36	1.48	< 0.001*	4.58	2.21	1.69	1.73	< 0.001*
L1-NB (mm)	9.61	1.93	5.68	1.60	< 0.001*	10.93	1.55	6.69	1.65	< 0.001*
U1-PP (mm)	30.70	2.80	29.15	2.78	0.005*	30.25	2.56	28.32	3.53	0.012*
U6-PP (mm)	20.21	2.48	19.85	1.97	0.254	19.98	2.59	18.42	2.70	< 0.001*
L1-MP (mm)	42.96	3.25	41.52	3.11	0.004*	43.48	2.34	41.52	3.06	0.001*
L6-MP (mm)	32.65	3.10	34.11	3.13	< 0.001*	31.88	2.66	34.27	3.12	< 0.001*
Occlusal Plane(°)	12.85	3.64	14.11	3.68	0.191	12.18	4.56	14.69	4.98	0.009*

**Table 7:** Comparison of changes in dental variables through cephalometric analysis between the MMA and MMA + VC groups

Variables	MMA (*N* = 17)		MMA + VC (*N* = 19)		*P* value
	Mean	SD	Mean	SD	
U1 - NA (°)	-5.59	8.69	-5.54	5.99	0.983
U1 - SN (°)	-7.14	8.58	-7.96	5.69	0.736
L1 - NB (°)	-8.72	5.67	-7.63	7.61	0.625
IMPA (°)	-8.73	5.67	-6.00	7.88	0.246
Inter incisor angle (U1-L1) (°)	15.91	10.40	15.55	7.98	0.909
U1-NA (mm)	-3.12	2.72	-2.89	1.87	0.774
L1-NB (mm)	-3.92	1.78	-4.24	1.92	0.616
U1-PP (mm)	-1.55	1.95	-1.93	3.03	0.658
U6-PP (mm)	-0.35	1.23	-1.56	1.52	0.013*
L1-MP (mm)	-1.44	1.79	-1.96	2.07	0.428
L6-MP (mm)	1.46	1.28	2.38	1.94	0.104
Occlusal Plane(°)	1.26	4.23	2.51	3.76	0.356

**Table 8:** Pre- and post-treatment comparison of sagittal and vertical tooth movements through model analysis between the TAD and MMA + VC groups

Variables	MMA (*N* = 17)		MMA + VC (*N* = 19)		*P*
	Mean	SD	Mean	SD	
Sagittal					
U1	5.87	2.03	6.33	1.53	0.455
U3	6.12	1.77	5.73	1.72	0.513
U6	-1.33	1.33	-1.10	1.22	0.587
Vertical					
U1	0.67	2.09	-1.30	1.61	0.004*
U3	0.33	1.78	-1.81	1.28	< 0.001*
U6	-0.86	0.89	-2.25	1.03	< 0.001*

### Multivariate regression analysis revealed the key factors of profile improvement

Since the changes occurred at the same time, assessing which factors played the most important role in altering the patients’ soft tissue profile was difficult. Therefore, we selected the Z angle—one of the most representative and remarkably changed profile indicators—as the dependent variable for our analysis, and we conducted multiple linear regression of the standardized bone, tooth, and soft tissue measurements.

Considering the interference of collinearity, we selected the following representative indicators: ANB, MP-SN, PFH/AFH, U1/SN, IMPA, U1-PP, U6-PP, L1-MP, L6-MP, Pog-NB, UL thickness, LL thickness, and Chin thickness.

The results showed that *Y* = 0.000576 − 0.416*a* − 0.340*b* + 0.403*c* (where *Y* denotes the Z angle and *a*, *b*, and *c* represent the MP-SN, U1-SN, and Pog-NB, respectively; Table 9). This finding indicated that the change in the Z angle was negatively correlated with the MP-SN and U1-SN variables and positively correlated with Pog-NB.

**Table 9:** Correlation between the change in variables for the craniofacial structures and the Z angle tested via multivariable linear regression analysis

Dependent variables	β	*P*
MP-SN	-0.416	0.009*
U1-SN	-0.340	0.030*
Pog-NB	0.403	0.008*

Thus, the gratifying profile improvement of patients with skeletal class II and the hyperdivergent facial type relied on the massive retraction of the upper incisors, the shape of the chin, and the mandibular plane’s counterclockwise rotation.

## Discussion

### The efficacy of TAD-assisted vertical control

In this retrospective study, we endeavored to quantify the efficacy of TAD-facilitated vertical control in managing maxillary dental intrusion and consequent mandibular counterclockwise rotation. Subsequently, we elucidated their pivotal roles in enhancing soft tissue profiles according to the baseline of MMA group.

Evaluation of hard tissue showed that following en-masse retraction with mini-implants anchorage, the MMA group exhibited slight upper molar intrusion (U6: -0.86 ± 0.89 mm) and mandibular counterclockwise rotation (MP-SN: -0.16 ± 1.05°). This result is consistent with the randomized controlled trial conducted by Al-Sibale et al. [25] and the controlled clinical trial conducted by Chen et al. [14], suggesting that TADs in the maxillary alveolar can provide some vertical force even during sagittal retraction, necessitating attention to the direction of traction and the vertical position of the anterior teeth to avoid deepening of the overbite. Following active maxillary dental intrusion, the MMA + VC group exhibited greater upper molar intrusion (U6: -2.25 ± 1.03 mm) and mandibular counterclockwise rotation (MP-SN: -1.82 ± 1.38°), which is slightly lower than that reported by Ding et al. [15] and Deguchi et al. [26] This difference may be attributed to differences in inclusion criteria. In Ding’s study, the inclusion criteria were shallow overbite, while in Deguchi’s study were open bite. In contrast, our study included many patients with normal or even deep overbite. To achieve a favorable overbite after treatment, we conducted intrusion of not only molars but also anterior teeth (U1: -1.30 ± 1.61 mm; U3: -1.81 ± 1.28 mm) with the help of TADs in the anterior segment, which represented a more challenging improvement compared to the aforementioned studies.

In terms of soft tissue evaluation, many previous studies have discussed the main factors contributing to changes in various soft tissue landmarks. For instance, Maetevorakul et al. found that the improvement in incisor angle was most crucial for enhancing lower lip prominence, and the mandibular plane angle as well as different treatment modalities had significant effects on changes of soft tissue chin prominence [27]. Regarding the overall assessment of soft tissue profiles, Zhao et al. demonstrated that the Z angle had the best discriminative ability for female adults with Angle Class II Division 1 malocclusion [28]. Therefore, in this study, we stressed on the Z angle and found that the MMA + VC group showed a more significant improvement compared to the MMA group (15.25 ± 5.30° in the MMA + VC group; 10.54 ± 5.11° in the MMA group, *P* = 0.011), which correlates with the poorer profiles before treatment in the MMA + VC group. To better identify which patients require active dental intrusion, we conducted a multiple linear regression analysis and found that this improvement was most closely associated with the retraction of the upper anterior teeth, prominence of the pogonion, and counterclockwise rotation of the mandibular plane. Therefore, we can conclude that vertical control is more necessary for patients with limited space for retraction or poor chin morphology.

### Limitations and prospects of TAD-assisted vertical control

Although the occlusal plane’s counterclockwise rotation is considered an effective method to reduce the angle of the mandibular plane [29], in the current study, we observed a trend of clockwise rotation. However, this unexpected result is consistent with the findings of many similar studies in this field [12, 13]. We speculate that this rotation results from the pendulum effect of the upper anterior teeth. Compared with the molars, the upper incisors have less intrusion, suggesting that we must pay particular attention to controlling the occlusal plane.

Additionally, despite TADs’ advantages of simplicity, flexibility, and independence from patient cooperation, they remain an invasive treatment [30, 31]. In the current study, however, six miniscrews were needed to achieve effective vertical control. This approach does not apply to patients with improper bone conditions, and it also increases the difficulty of operation. Therefore, we hope to develop further methods that are more convenient and minimally invasive. The use of midpalatal miniscrews and personalized palatal bars may be an alternative option [12]; however, such an approach would still pose challenges in terms of operation and hygiene maintenance. Accordingly, we hope to further reduce orthodontic devices’ complexity in order to meet the requirements of comfortable treatment.

Methodologically, the current study’s evaluation of muscle response and profile changes was limited to a cephalometric analysis. Since soft tissue yields inaccurate measurements during lateral cephalograms, 3D facial scanning and electromyography could allow a more precise examination of patients’ aesthetic and functional changes. We plan to enhance the refinement of assessment modalities for both soft and hard tissues, endeavoring to substantiate vertical control’s efficacy and constraints through various methodologies, including randomized controlled trials.

## Conclusions

The conclusions of this retrospective study are as follows.

- TAD-reinforced maxillary anchorage with vertical control achieves intrusion of approximately 2 mm for the upper first molars.
- TAD-reinforced maxillary anchorage with vertical control induces mandibular counterclockwise rotation of approximately 1.8° and improves patients’ hyperdivergent skeletal pattern.
- When the upper incisors are not sufficiently retracted or the chin shape is not satisfying, active vertical control should be applied to help patients achieve better profiles.

Taken together, these conclusions demonstrate that TAD-assisted vertical control is essential for patients with skeletal class II and a hyperdivergent facial type. This approach constitutes a good alternative to improving occlusion and profiles via orthodontic camouflage treatment.

### Electronic supplementary material

Below is the link to the electronic supplementary material.

Supplementary Material 1

## Data Availability

The data sets used and analyzed during the current study are available from the corresponding author on reasonable request.

## Abbreviations

MMA: Maxillary maximum anchorage
TAD: Temporary anchorage devices
VC: Vertical control

## References

[1] Tucker MR (1995). Orthognathic surgery versus orthodontic camouflage in the treatment of mandibular deficiency. J Oral Maxillofac Surg.

[2] Baherimoghaddam T, Oshagh M, Naseri N, Nasrbadi NI, Torkan S (2014). Changes in cephalometric variables after orthognathic surgery and their relationship to patients’ quality of life and satisfaction. J Oral Maxillofac Res.

[3] Thomas PM (1995). Orthodontic camouflage versus orthognathic surgery in the treatment of mandibular deficiency. J Oral Maxillofac Surg.

[4] Raposo R, Peleteiro B, Paco M, Pinho T (2018). Orthodontic camouflage versus orthodontic-orthognathic surgical treatment in class II malocclusion: a systematic review and meta-analysis. Int J Oral Maxillofac Surg.

[5] Freitas MR, Lima DV, Freitas KM, Janson G, Henriques JF (2008). Cephalometric evaluation of class II malocclusion treatment with cervical headgear and mandibular fixed appliances. Eur J Orthod.

[6] Jung MH (2019). Vertical control of a class II deep bite malocclusion with the use of orthodontic mini-implants. Am J Orthod Dentofac Orthop.

[7] Peng J, Lei Y, Liu Y, Zhang B, Chen J (2023). Effectiveness of micro-implant in vertical control during orthodontic extraction treatment in class II adults and adolescents after pubertal growth peak: a systematic review and meta-analysis. Clin Oral Investig.

[8] Jamilian A, Showkatbakhsh R, Rad AT (2012). A novel approach for treatment of mandibular deficiency with vertical growth pattern. Int J Orthod Milwaukee.

[9] Lione R, Franchi L, Laganà G, Cozza P (2015). Effects of cervical headgear and pendulum appliance on vertical dimension in growing subjects: a retrospective controlled clinical trial. Eur J Orthod.

[10] Sambataro S, Rossi O, Bocchieri S, Fastuca R, Oppermann N, Levrini L (2023). Comparison of cephalometric changes in Class II growing patients with increased vertical dimension after high-pull and cervical headgear treatment. Eur J Paediatr Dent.

[11] Ulger G, Arun T, Sayinsu K, Isik F (2006). The role of cervical headgear and lower utility arch in the control of the vertical dimension. Am J Orthod Dentofac Orthop.

[12] Lee J, Miyazawa K, Tabuchi M, Kawaguchi M, Shibata M, Goto S (2013). Midpalatal miniscrews and high-pull headgear for anteroposterior and vertical anchorage control: cephalometric comparisons of treatment changes. Am J Orthod Dentofac Orthop.

[13] Yao CC, Lai EH, Chang JZ, Chen I, Chen YJ (2008). Comparison of treatment outcomes between skeletal anchorage and extraoral anchorage in adults with maxillary dentoalveolar protrusion. Am J Orthod Dentofac Orthop.

[14] Chen M, Li ZM, Liu X, Cai B, Wang DW, Feng ZC (2015). Differences of treatment outcomes between self-ligating brackets with microimplant and headgear anchorages in adults with bimaxillary protrusion. Am J Orthod Dentofac Orthop.

[15] Ding S, Liu M, Zou T (2019). Comparative study on vertical effect between miniscrew and face-bow in orthodontic treatment of hyperdivergent class II protrusion. J Oral Sci Res.

[16] Khlef HN, Hajeer MY, Ajaj MA, Heshmeh O (2018). Evaluation of treatment outcomes of En masse Retraction with Temporary skeletal Anchorage devices in comparison with two-step retraction with Conventional Anchorage in patients with Dentoalveolar Protrusion: a systematic review and Meta-analysis. Contemp Clin Dent.

[17] Kokodynski RA, Marshall SD, Ayer W, Weintraub NH, Hoffman DL (1997). Profile changes associated with maxillary incisor retraction in the postadolescent orthodontic patient. Int J Adult Orthodon Orthognath Surg.

[18] Kuroda S, Yamada K, Deguchi T, Kyung HM, Takano-Yamamoto T (2009). Class II malocclusion treated with miniscrew anchorage: comparison with traditional orthodontic mechanics outcomes. Am J Orthod Dentofac Orthop.

[19] Wang XD, Zhang JN, Liu DW, Lei FF, Liu WT, Song Y (2017). Nonsurgical correction using miniscrew-assisted vertical control of a severe high angle with mandibular retrusion and gummy smile in an adult. Am J Orthod Dentofac Orthop.

[20] Deng JR, Li YA, Wang XD, Li J, Ding Y, Zhou YH (2018). Evaluation of Long-Term Stability of Vertical Control in Hyperdivergent patients treated with Temporary Anchorage devices. Curr Med Sci.

[21] Wang XD, Zhang JN, Liu DW, Lei FF, Zhou YH (2016). Nonsurgical correction of a severe anterior deep overbite accompanied by a gummy smile and posterior scissor bite using a miniscrew-assisted straight-wire technique in an adult high-angle case. Korean J Orthod.

[22] Wang XD, Lei FF, Liu DW, Zhang JN, Liu WT, Song Y (2017). Miniscrew-assisted customized lingual appliances for predictable treatment of skeletal class II malocclusion with severe deep overbite and overjet. Am J Orthod Dentofac Orthop.

[23] Wang Y, Zhou Y, Zhang J, Wang X (2022). Long-term stability of counterclockwise mandibular rotation by miniscrew-assisted maxillary intrusion in adult patients with skeletal class II high-angle malocclusion: a 10-year follow-up of 2 patients. AJO-DO Clin Companion.

[24] Haralabakis NB, Sifakakis IB (2004). The effect of cervical headgear on patients with high or low mandibular plane angles and the myth of posterior mandibular rotation. Am J Orthod Dentofac Orthop.

[25] Al-Sibaie S, Hajeer MY (2014). Assessment of changes following en-masse retraction with mini-implants anchorage compared to two-step retraction with conventional anchorage in patients with class II division 1 malocclusion: a randomized controlled trial. Eur J Orthod.

[26] Deguchi T, Kurosaka H, Oikawa H, Kuroda S, Takahashi I, Yamashiro T (2011). Comparison of orthodontic treatment outcomes in adults with skeletal open bite between conventional edgewise treatment and implant-anchored orthodontics. Am J Orthod Dentofac Orthop.

[27] Maetevorakul S, Viteporn S (2016). Factors influencing soft tissue profile changes following orthodontic treatment in patients with Class II Division 1 malocclusion. Prog Orthod.

[28] Zhao Z (2016). The study on sensitivity of Asethetic Index from six kinds of Profile Soft tissue analysis methods on female adult in Liaoning Province.

[29] Li X, Zhao Q, Zhao R, Gao M, Gao X, Lai W (2017). Effect of occlusal plane control procedure on hyoid bone position and pharyngeal airway of hyperdivergent skeletal class II patients. Angle Orthod.

[30] Papadopoulos MA, Papageorgiou SN, Zogakis IP (2011). Clinical effectiveness of orthodontic miniscrew implants: a meta-analysis. J Dent Res.

[31] Yamaguchi M, Inami T, Ito K, Kasai K, Tanimoto Y (2012). Mini-implants in the anchorage armamentarium: new paradigms in the orthodontics. Int J Biomater.

