# Supplementary material for: Quantitative evaluation of vertical control in orthodontic camouflage treatment for skeletal class II with hyperdivergent facial type
Source: Head Face Med. 2024 May 14;20:31. doi: 10.1186/s13005-024-00432-2 (PMC11092056; doi:10.1186/s13005-024-00432-2)
Supplement: Supplementary file 1 — Supplementary Material 1 [file 13005_2024_432_MOESM1_ESM.docx]

**Additional Tables**

Additional Table 1. Age and treatment duration of MMA and MMA+VC groups.

|  | MMA (N=17) | | MMA+VC (N=19) | | *P* |
| --- | --- | --- | --- | --- | --- |
|  | Mean | SD | Mean | SD |  |
| Age (years) | 24.18 | 3.83 | 25.00 | 4.99 | 0.586 |
| Treatment duration (months) | 34.4 | 12.8 | 34.7 | 6.8 | 0.512 |

| Abbreviation | Definition |
| --- | --- |
| S | Sella: the center of the hypophyseal fossa. |
| N | Nasion: the most anterior point of the frontonasal suture in the median plane. |
| A | Point A: the point at the deepest midline concavity on the maxilla between the anterior nasal spine and prosthion. |
| B | Point B: the point at the deepest midline concavity on the mandibular symphysis between infradentale and pogonion. |
| Pog | Pogonion: the most anterior point of mandibular symphysis. |
| ANS | Anterior nasal spine: the tip of the bony anterior nasal spine. |
| Me | Menton: the most inferior point on the symphysis of the mandible. |
| Go | Constructed gonion: bisecting the angle formed by the tangents to the lower and the posterior borders of the mandible. |
| U1 | Maxillary central incisor: the most labial point on the crown of maxillary central incisor. |
| L1 | Mandibular central incisor: the most labial point on the crown of mandibular central incisor. |
| U6 | Maxillary first molar: the tip of the mesiobuccal cusp of the maxillary first molar. |
| L6 | Mandibular first molar: the tip of the mesiobuccal cusp of the mandibular first molar. |
| UL | Upper Lip: the most anterior point of the upper lip. |
| LL | Lower Lip: the most anterior point of the lower lip. |
| LFH | Lower Facial Height: the distance between point ANS and Me. |
| TFH | Total Facial Height: the sum of the distance of N-ANS and ANS-Me. |
| PFH | Posterior Facial Height: the distance between point S and Go. |
| AFH | Anterior Facial Height: the distance between point N and Me. |
| UL thickness | Upper Lip Thickness: the distance between point UL and U1. |
| LL Thickness | Lower Lip Thickness: the distance between point B and the deepest concavity on the soft tissue outline between point LL to the most anterior point of chin. |
| UL Length | Upper Lip Length: the distance between subnasale to stomion superius. |
| LL Length | Lower Lip Length: the distance between stomion inferius to the most inferior point of chin. |
| MP | Mandibular Plane: the line connecting the point Go to Me. |
| PP | Palatal Plane: the line joining anterior nasal spine with posterior nasal spine. |
| OP | Occlusal Plane: the line connecting the tip of U1 and mesiobuccal cusp of U6. |
| FH | Frankfort Horizontal Plane: the line connecting the orbitale and porion. |
| SnV | Sn Vertical Line: the vertical line pass through the subnasale. |
| FMA | Angle between MP and FH. |
| IMPA | Angle between the long axis of L1 and MP. |
| UL Angle-SnV | Angle between SnV and the line connecting Subnasale and UL. |
| Z Angle | Angle between FH and the line connecting the most protrusive point of lip and the most anterior point of chin. |

Additional Table 2. The definition of landmarks and measurements.

Additional Table 3. The results of ICC test showed good consistency of the measurements.

| Variables | α | *P* |
| --- | --- | --- |
| Skeletal | | |
| SNA (°) | 0.946 | < 0.001* |
| SNB (°) | 0.966 | < 0.001* |
| ANB (°) | 0.867 | 0.003* |
| MP-SN (°) | 0.955 | < 0.001* |
| FMA (°) | 0.929 | < 0.001* |
| LFH/TFH (%) | 0.933 | < 0.001* |
| PFH/AFH (%) | 0.959 | < 0.001* |
| Pog-NB (mm) | 0.855 | 0.004* |
| ANS-Me (mm) | 0.823 | 0.008* |
| Dental | | |
| U1 - NA (°) | 0.967 | < 0.001* |
| U1 - SN (°) | 0.969 | < 0.001* |
| L1 - NB (°) | 0.887 | 0.002* |
| IMPA (°) | 0.831 | 0.007* |
| Inter incisor angle (U1-L1) (°) | 0.940 | < 0.001* |
| U1-NA (mm) | 0.944 | < 0.001* |
| L1-NB (mm) | 0.858 | 0.004* |
| U1-PP (mm) | 0.870 | 0.003* |
| U6-PP (mm) | 0.938 | < 0.001* |
| L1-MP (mm) | 0.835 | 0.005* |
| L6-MP (mm) | 0.909 | 0.001* |
| Soft tissue related | | |
| UL Angle-SnV (°) | 0.983 | < 0.001* |
| Z Angle (°) | 0.937 | < 0.001* |
| UL thickness (mm) | 0.844 | 0.005* |
| LL Thickness (mm) | 0.924 | < 0.001* |
| Pog-Pog' (mm) | 0.810 | 0.011* |
| UL-Sn Vertical (mm) | 0.867 | 0.003* |
| LL-Sn Vertical (mm) | 0.923 | < 0.001* |
| UL Length (mm) | 0.894 | 0.001* |
| LL Length (mm) | 0.973 | < 0.001* |
| Digital casts superimposition | | |
| Sagittal-U1 | 0.997 | < 0.001* |
| Sagittal-U3 | 0.981 | < 0.001* |
| Sagittal-U6 | 0.914 | < 0.001* |
| Vertical-U1 | 0.993 | < 0.001* |
| Vertical-U3 | 0.993 | < 0.001* |
| Vertical-U6 | 0.997 | < 0.001* |
